# Cardiac arrhythmogenesis: roles of ion channels and their functional modification

**DOI:** 10.3389/fphys.2024.1342761

**Published:** 2024-03-04

**Authors:** Ming Lei, Samantha C. Salvage, Antony P. Jackson, Christopher L.-H. Huang

**Affiliations:** ^1^ Department of Pharmacology, University of Oxford, Oxford, United Kingdom; ^2^ Department of Biochemistry, University of Cambridge, Cambridge, United Kingdom; ^3^ Physiological Laboratory, University of Cambridge, Cambridge, United Kingdom

**Keywords:** cardiac arrhythmias, ion channels, excitation–contraction coupling, drug discovery, cardiac remodeling

## Abstract

Cardiac arrhythmias cause significant morbidity and mortality and pose a major public health problem. They arise from disruptions in the normally orderly propagation of cardiac electrophysiological activation and recovery through successive cardiomyocytes in the heart. They reflect abnormalities in automaticity, initiation, conduction, or recovery in cardiomyocyte excitation. The latter properties are dependent on surface membrane electrophysiological mechanisms underlying the cardiac action potential. Their disruption results from spatial or temporal instabilities and heterogeneities in the generation and propagation of cellular excitation. These arise from abnormal function in their underlying surface membrane, ion channels, and transporters, as well as the interactions between them. The latter, in turn, form common regulatory targets for the hierarchical network of diverse signaling mechanisms reviewed here. In addition to direct *molecular-level* pharmacological or physiological actions on these surface membrane biomolecules, accessory, adhesion, signal transduction, and cytoskeletal anchoring proteins modify both their properties and localization. At the *cellular* level of excitation–contraction coupling processes, Ca^2+^ homeostatic and phosphorylation processes affect channel activity and membrane excitability directly or through intermediate signaling. *Systems*-level autonomic cellular signaling exerts both acute channel and longer-term actions on channel expression. Further upstream intermediaries from metabolic changes modulate the channels both themselves and through modifying Ca^2+^ homeostasis. Finally, longer-term *organ*-level inflammatory and structural changes, such as fibrotic and hypertrophic remodeling, similarly can influence all these physiological processes with potential pro-arrhythmic consequences. These normal physiological processes may target either individual or groups of ionic channel species and alter with particular pathological conditions. They are also potentially alterable by direct pharmacological action, or effects on longer-term targets modifying protein or cofactor structure, expression, or localization. Their participating specific biomolecules, often clarified in experimental genetically modified models, thus constitute potential therapeutic targets. The insights clarified by the physiological and pharmacological framework outlined here provide a basis for a recent modernized drug classification. Together, they offer a translational framework for current drug understanding. This would facilitate future mechanistically directed therapeutic advances, for which a number of examples are considered here. The latter are potentially useful for treating cardiac, in particular arrhythmic, disease.

## 1 Introduction: normal and abnormal cardiac rhythms

### 1.1 Clinical importance of cardiac arrhythmias

**FIGURE 1:**
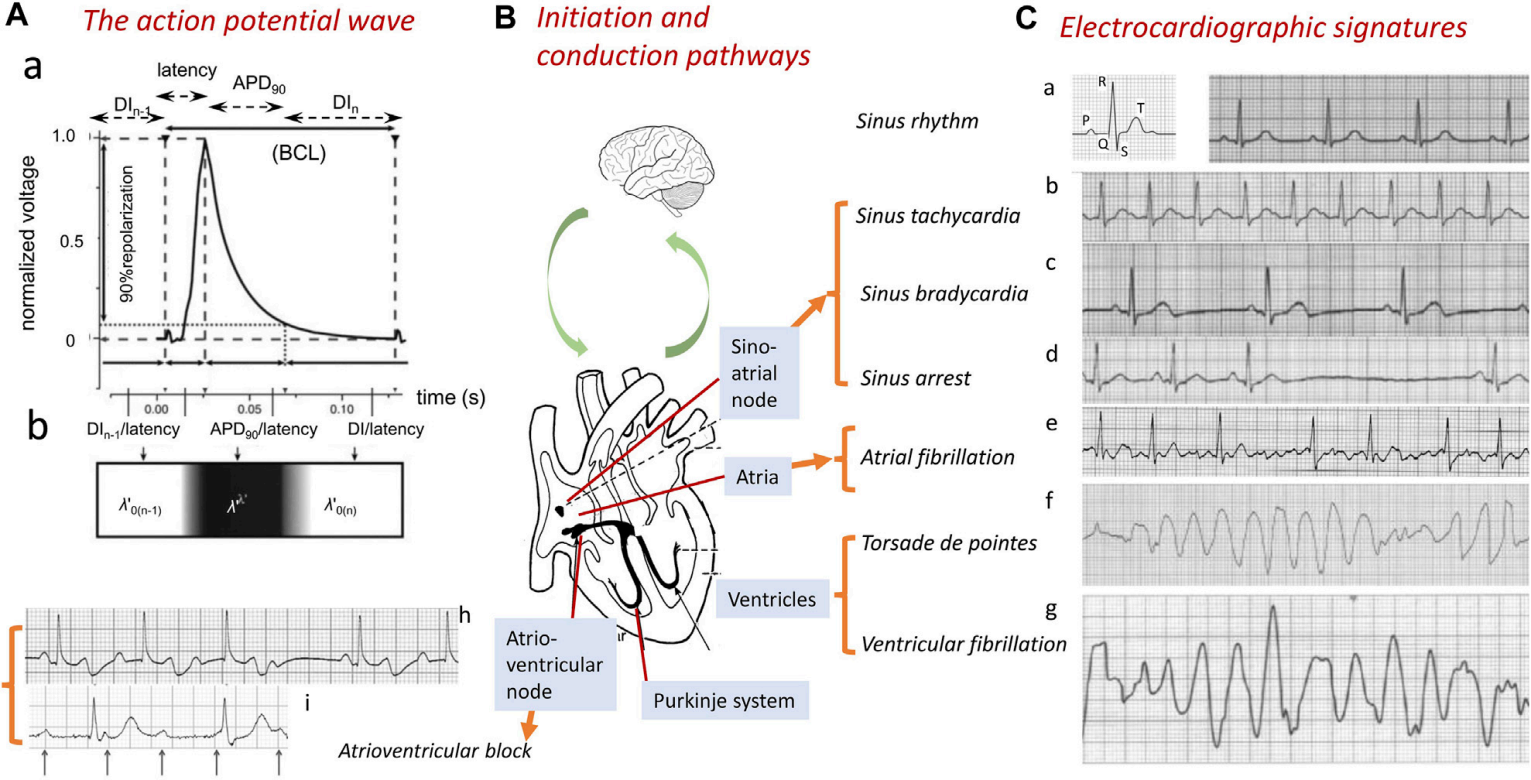
Normal and abnormal cardiac conduction. Generation of the normal wave of cardiac excitation, exemplified by **(A)** a propagating action potential (AP) in the right ventricle of a murine heart beating at 600 bpm. The AP comprises a depolarization phase following a conduction latency, followed by repolarization recovery and a refractory period (a). The AP waveform can be characterized by its BCL, APD_90_, latency, and the DI of the current (*n*th) and preceding [(*n*-1)^th^] action potential. The latter spatially map onto a traveling wave characterized by its conduction velocity *θ* and active and resting wavelengths *λ*′ and *λ*_0_’ and basic cycle distance, BCD’ = *λ*’ + *λ*_0_’ (b). The AP is **(B)** transmitted through successive initiating, sino-atrial node (SAN), conducting, atrioventricular (AV) and Purkinje, tissue, and contractile atrial and ventricular structures. These processes are subject to modulation at the systems, including the nervous system level. **(C)** Abnormalities in this communication disrupt the normal cardiac electrocardiographic signal (a), resulting in abnormal electrical patterns. These are exemplified by sinus rhythm tachy- or bradycardic abnormalities (a–c) or arrest (d) and breakdown in orderly excitation resulting in atrial fibrillation (e) or ventricular torsade de pointes (f) or fibrillation (g). AVN or Purkinje tissue conduction block is exemplified here by second-degree Mobitz I heart block (h) and third-degree complete heart block (i) [**(A)** from Figure 1 of the work of Matthews et al. (2013); **(C)** adapted from Figure 14.1 of the work of Huang (2021)].

The co-ordinated and effective mechanical function of the heart requires orderly initiation and conduction of electrophysiological action potential (AP, Figure 1A) signals through its participating excitable cells. This process involves successively the atria, atrioventricular node (AVN), Purkinje conducting tissue, and ventricular endocardial and epicardial myocardium (Figure 1B). The breakdown of this process constitutes the underlying cause of cardiac arrhythmias, the major contributors to clinical mortality and morbidity. Autopsies of 5–35-year-old patients implicate cardiac causes in 56.4% of non-traumatic sudden deaths. Of these, ∼30% may be arrhythmic, with many stemming from ischemic heart disease (Behr et al., 2003). However, ∼4% of sudden cardiac deaths (SCDs) show no accompanying structural abnormalities (Tung et al., 1994; Clancy and Kass, 2005; Martin et al., 2011d; Martin et al., 2012; Martin et al., 2013), implicating underlying channelopathy (Tester and Ackerman, 2007; Bezzina et al., 2015). Arrhythmias also arise as adverse effects of pharmacotherapeutic agents (Killeen, 2009).

Different arrhythmias show specific electrocardiographic (ECG) signatures varying with the cardiac region involved (Figure 1C) (Huang, 2021). S*inus node disorders* manifest as tachycardic conditions, as well as, contrastingly, sinus pauses/arrests, chronotropic incompetence, and other bradycardic conditions. The latter constitute indications for ∼50% of the 10^6^/y permanent pacemaker implants worldwide (de Marneffe et al., 1993; Dobrzynski et al., 2007; Dobrev, 2009). Requirements for their implantation increases with age, reaching 1:600 at age >65 years. The most common sustained arrhythmia, *atrial fibrillation* (AF), similarly age-related (Dun and Boyden, 2009; Wakili et al., 2011), affects 1%–2% of the general population (Stewart et al., 2002; Lloyd-Jones et al., 2004; Andrade et al., 2014), predisposing to further major cardiac and cerebrovascular morbidity and mortality (Stewart et al., 2002), including stroke (Wolf et al., 1991). *Ventricular arrhythmias*, exemplified by ventricular tachycardia (VT), potentially leading to ventricular fibrillation (VF) and SCD (Rubart and Zipes, 2005; Turakhia and Tseng, 2007), are implicated in >300,000 and ∼70,000 deaths/year in the United States (Kannel et al., 1987) and United Kingdom, respectively (Colquitt et al., 2014). Some degree of rhythmic cardiac activity persists with AVN or Purkinje conduction blocks. Of the different extents of AVN conduction block, first-degree block results in electrocardiographic (ECG) complexes showing consistent PR interval prolongations (>0.20 s). Second-degree blocks Mobitz type 1 (Wenckebach) and Mobitz type 2 both manifest as repeated ECG cycles with dropped QRS complexes, The latter are preceded by cycles showing progressively increasing (Figure 1Ch) and constant PR intervals, respectively. Third-degree heart block (Figure 1Ci) contrastingly manifests as complete P wave and QRS complex dissociation. Finally, Purkinje tissue right and left bundle branch blocks result in broadened (>120 ms duration) QRS complexes with abnormal kinetic features varying with right- or left-sided ECG leads and the site of the block.

### 1.2 Normal cardiac activity

**FIGURE 2:**
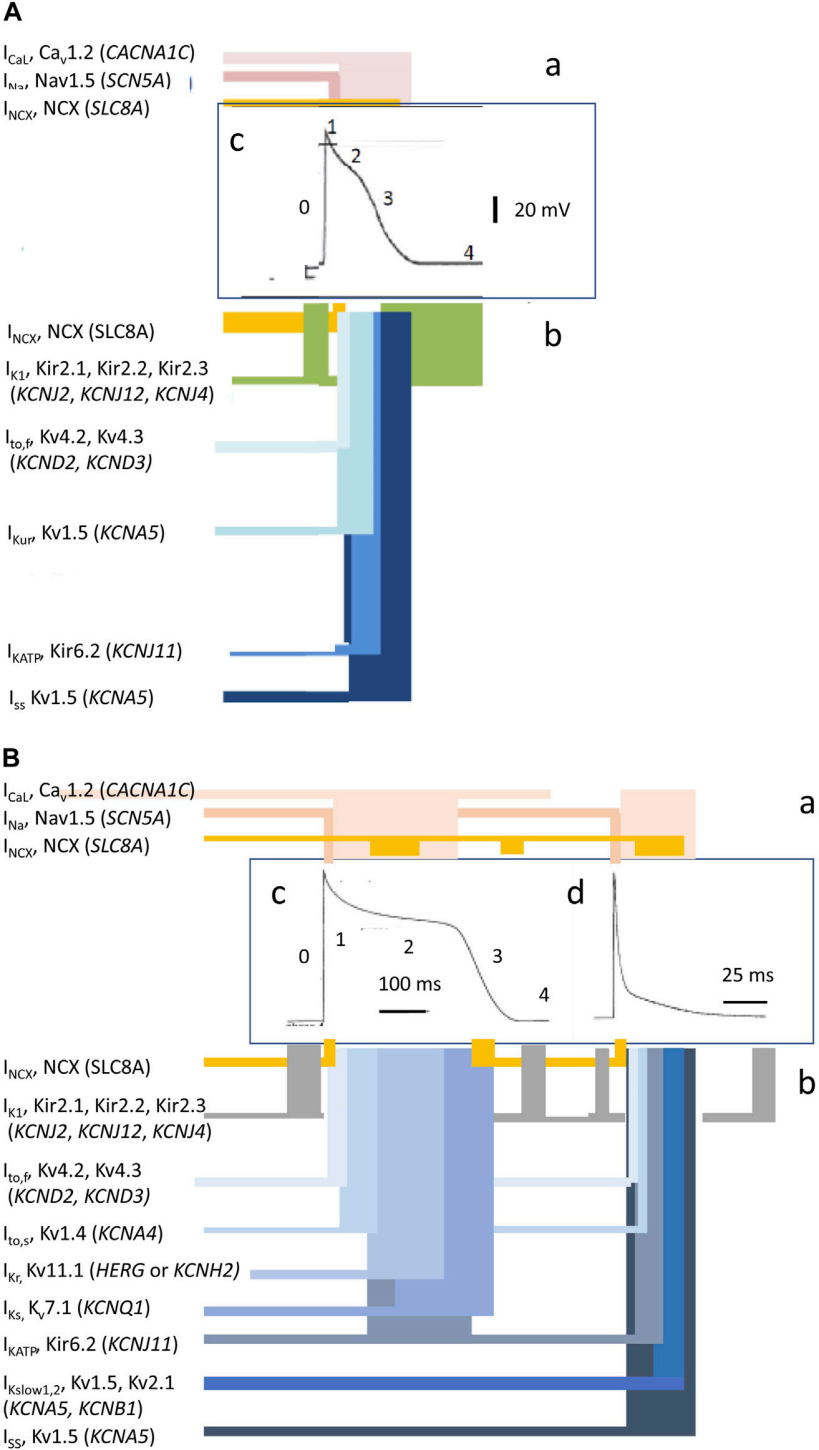
Ion current contributions to basic features of cardiac electrophysiological excitation. Human atrial **(A)** and human and murine ventricular **(B)** inward (a) and outward ionic current contributions (b), atiributable to surface membrane ion channels, to human (c) and mouse (d) AP waveforms [from Figure 1 of the work of Huang (2017) and Figure 1 of the work of Huang et al. (2020)].

Cardiac excitation is initiated and paced by repetitive cycles of sino-atrial node excitation (Lei et al., 2007; Donald and Lakatta, 2023). These trigger periodic waves of APs that then propagate through successive cardiac structures (Figure 1B) (Draper and Weidmann, 1951; Matthews et al., 2013). Each AP begins with rapid phase 0 depolarization from the resting membrane potential. Subsequent completion of the slower recovery over action potential duration (APD) and effective refractory period (ERP) permits further excitation cycles. *Atrial APs* show triangular waveforms with prominent initial phase 1 recoveries (Figure 2A). Human *ventricular APs* (Figure 2B) similarly begin with rapid (∼400 V s^−1^) phase 0 depolarization from the (∼-90 mV) resting potential to a +40 to +60 mV overshoot voltage. The subsequent phase 1 initial rapid repolarization is followed by a prolonged phase 2 plateau, lengthening the APD and ERP. It is terminated by phase 3 repolarization leading to phase 4 electrical diastole (Weidmann, 1951). APs initiate the physiologically important cellular events mediating cardiac systole/diastole. They also drive cell-to-cell axial currents through cytoplasmic and gap junction resistances. They also possibly activate cell–cell ephaptic couplings. The latter events enable their propagation into hitherto quiescent cells, forming a coherent, advancing electrical wavefront through conducting or myocardial tissue, while leaving a trailing, recovering refractory region. Such excitation wavefronts span the thickness of the atrial wall. Ventricular propagation involves excitation proceeding from the transmural epicardium to endocardium and from the base to apex.

## 2 Electrophysiological basis of arrhythmogenesis

### 2.1 Spatial stability and instability in the propagated AP wave

This normal propagation of activation waves through continuous electrically coupled three-dimensional cardiomyocyte networks (Kucera et al., 1998; Kléber and Rudy, 2004) can be disrupted by pro-arrhythmic ectopic *triggering events.* In contrast, compromised spatial and temporal coherence and stability in the wavefronts generate an *arrhythmic substrate*. The *spatial extent and stability of* this *excitation* depend on its wavelength, *λ*, within which further premature excitation cannot occur. This normally ensures that only one such generated and propagated wave can occur per heartbeat. The term λ is determined by the propagation velocity *θ* and recovery ERP or APD: *λ* = *θ×*ERP or *θ×*APD (Huang, 2017). Figure 1Aa illustrates a typical basic cycle length (BCL) containing a murine ventricular AP. It defines its critical *temporal* properties of latency, APD, and succeeding diastolic interval, DI_n_, at 90% repolarization (APD_90_ and DI_90_). It then maps these *temporal* properties onto their corresponding *spatial* properties, assuming a constant AP conduction velocity *θ*. In the cardiac tissue through which the AP propagates, the latter form active and resting wavelengths λ′ and λ_0_’, making up the basic cycle distance, BCD’ = λ’ + λ_0_’, of the propagated AP waveform (Figure 1Ab).

**FIGURE 3:**
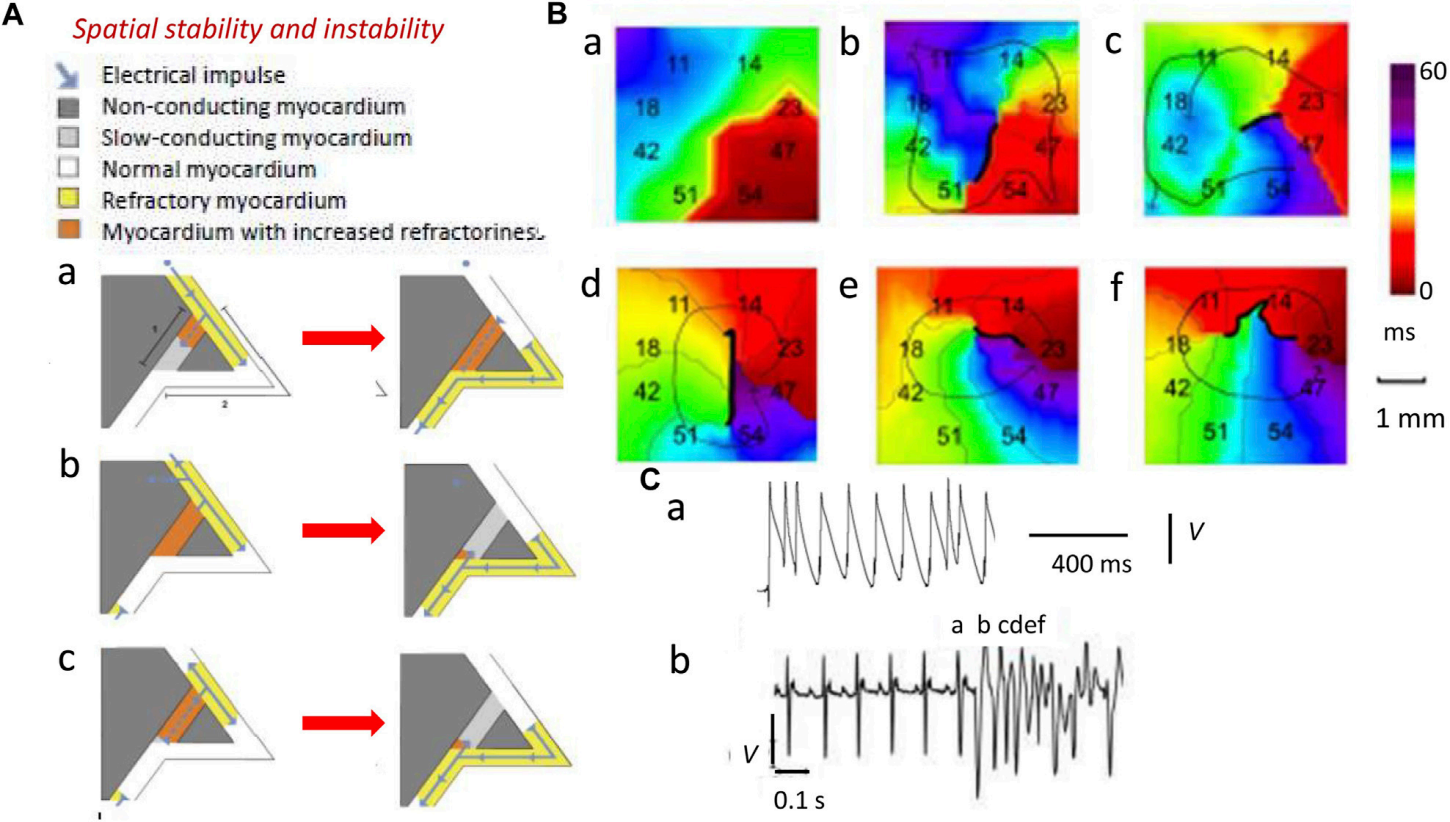
Spatial instabilities in the genesis of re-entrant arrhythmia. **(A)** Pro-arrhythmic mechanisms involving the conducting pathway with compromised conduction [dark gray path 1, (a)] and a second normally conducting pathway [white path 2, (a)]. A normal AP conduction with velocity *θ* and ERP has wavelength *λ* = θ × ERP (yellow region) in path 2 (b) (blue arrow). If the latter initiates a slow-conducting AP, this travels along path 1 (a) but under normal circumstances (b), this collides with refractory tissue within path 2 and then cannot re-enter the circuit (b). Thus, an abnormal triggered impulse immediately following the normal AP (a) cannot enter the refractory path 2 (b). However, a retrogradely conducting AP with reduced wavelength Λ shorter than the dimensions of the propagation pathways, due to reduced *θ* and/or ERP, along path 1 (a) entering the beginning of path 2 (b) causes self-perpetuating re-entrant excitation (c). **(B)** Isochronal AP propagation multi-electrode array mapping of re-entrant circuit initiation of ventricular tachycardia (VT) in the right ventricular epicardium of isolated Langendorff-perfused murine Scn5a+/− hearts following flecainide challenge. Thick black lines denote conduction block. Thin arrows denote lines of propagation. (a) Crowded isochronal lines in the last sinus beat, demonstrating area of conduction slowing. (b) Superimposed premature ventricular beat leads to line of block with impulse propagation flowing around it. (c) A second ventricular ectopic event causes the formation of a re-entrant circuit. (d) The circuit continues into the next beat (e, f) to initiate VT. Migration of line of block causing non-stationary vortex resulting in polymorphic arrhythmia should be noted. **(C)** Premature ventricular ectopic beat occurring as (a) isolated monophasically recorded event and (b) resulting in the initiation of electrocardiographically recorded sustained polymorphic VT [**(A)** Reproduced from King et al. (2013a), Figure 3; licensed under CC-BY 4.0 **(B)**, (Cb) Adapted from Martin et al. (2011c), Figure 4; (Ca) Reproduced from Killeen et al. (2007), Figure 5C].

Larger *λ* reduces the likelihood that areas of depolarization and repolarization collide to produce obstacles causing unidirectional conduction blocks. In the latter event, APs have to take a slow conducting pathway to traverse this non-conducting myocardium. Such blocks result from *depolarization abnormalities* (Figure 3, path 1; dark gray) (King et al., 2013a). Collision would involve previously excited adjacent regions of the normal myocardium (path 2; white). However, should the latter possess APs with sufficient *λ* (yellow region), the collision would simply involve refractory tissue, precluding re-excitation in path 2 (Figure 3Aa). Similar outcomes result from impulses from ectopic triggering immediately following the normal AP (Figure 3Ab). The traveling wave then passes undisrupted over the heterogeneity (Weiss et al., 2005). Contrastingly, an AP conducting retrogradely along path 1 entering the beginning of path 2 with *λ* reduced to values smaller than the dimensions of the available circuits would initiate persistent re-entrant excitation into the recovered myocardial regions (Figure 3Bc) (King et al., 2013a; Huang, 2017).

Decreased λs, thus, increase likelihoods of regenerative wave breakup into multiple wavelets, forming scroll waves (Davidenko et al., 1995; Zaitsev et al., 2000; Pandit and Jalife, 2013) along chaotic conduction pathways (Krogh-Madsen et al., 2012; Matthews et al., 2013; Spector, 2013). Contact multi-electrode (0.5 mm) array isochronal AP mapping techniques visualized the initiation of such a sequence. These studies were performed in right ventricular (RV) epicardia of intact beating flecainide-challenged pro-arrhythmic murine *Scn5a*+/− hearts (Figures 3Ba–f) (Martin et al., 2011c). *Scn5a*+/− ventricle APs show slowed conduction and increased activation and recovery dispersions. Here, following a delayed epicardial activation in the last normal beat reflected in the close isochronal contours (Figure 3Ba) (Killeen et al., 2007), a superimposed premature ventricular activation (Figure 3Ca) produces a line of block with AP propagation flowing around it (Figure 3Bb). A ventricular ectopic event then initiates an anticlockwise running circuit (Figure 3Bc) persistent through the following beat (Figure 3Bd). A consequent continually changing line of block now produces a non-stationary vortex (Figures 3Be,f) causing the polymorphic VT, apparent in the accompanying electrocardiographic trace (Figure 3Cb).

In addition to *θ*, recovery ERPs and APDs also affect *λ* and wave stability (Kléber and Rudy, 2004; Sabir et al., 2008a; Killeen et al., 2008b). ERPs and APDs vary across normal mammalian ventricular myocardium, causing spatial, epicardium-to-endocardium, *transmural repolarization gradients*, preserving orderly electrophysiological activation/recovery sequences and reducing likelihoods of *repolarization abnormalities*. Finally, differences between ERP and APD leaving abnormal re-excitation windows shorten *λ*, predisposing to pro-arrhythmic ectopic events (Sabir et al., 2007; Ashino et al., 2010; Martin et al., 2011a).

### 2.2 Temporal stability and instability of the AP wave

**FIGURE 4:**
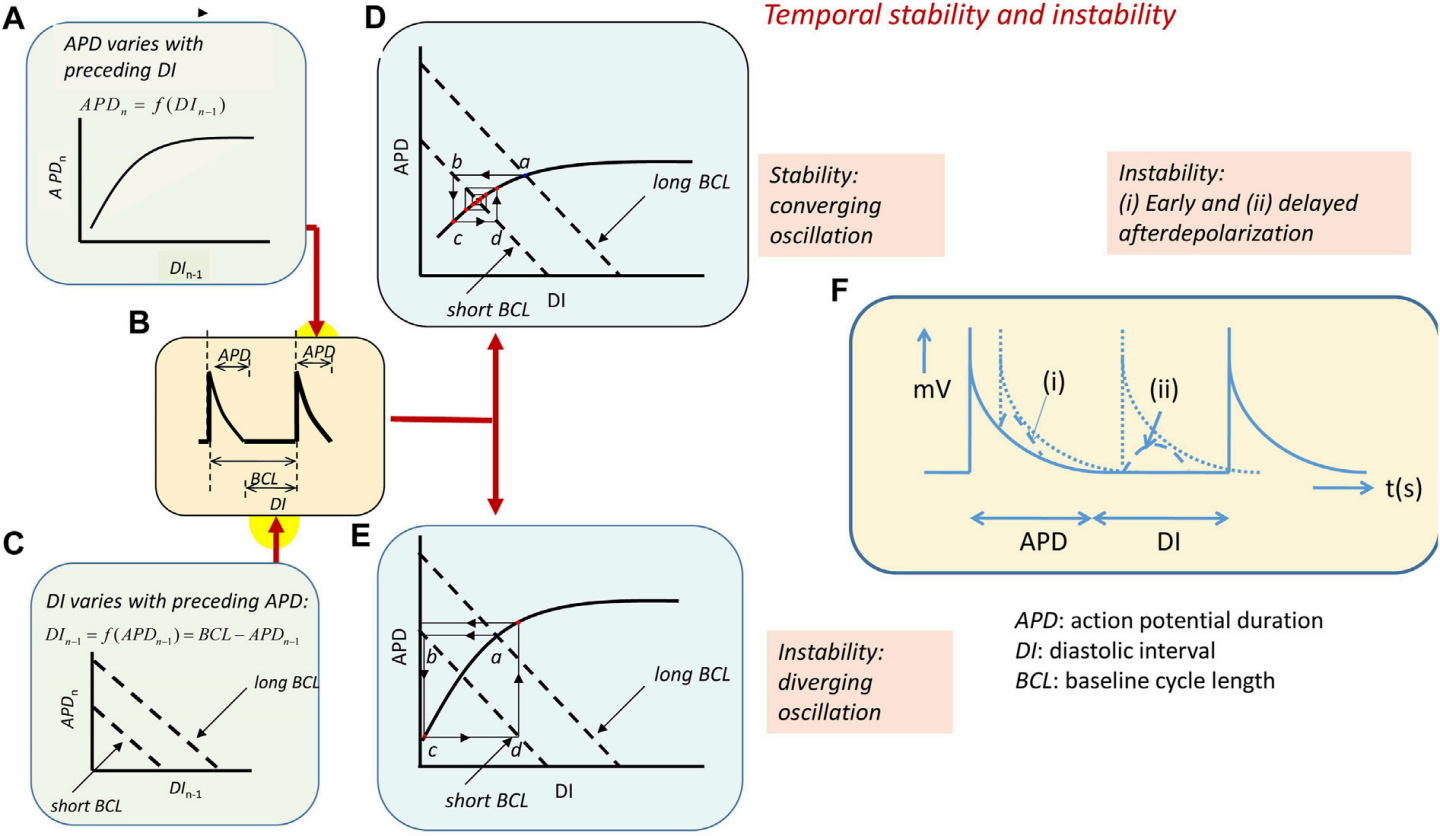
Arrhythmic substrate arising from temporal electrophysiological instabilities. **(A)** The *n*th action potential in a series has duration APD_n_ dependent on its preceding diastolic interval DI_n−1_ along an A-curve. Symbols clarified in **(B)** showing action potential with a BCL made up of the APD and succeeding diastolic interval DI. **(C)** D-line showing the linear effect of APD_n_ variations on DI_n−1_ at different BCLs: DI = BCL–APD. **(D, E)** Intersection between the A-curve and D-line gives steady-state APD and DI at any BCL (a). Increasing heart rate decreases the BCL and alters the D-line (b). This alters the APD (c) and, consequently, the succeeding DI (d). **(D)** A converging cycle resulting in declining oscillations results where APD_n_ depends on DI_n−1_ with less than unity slope. The cycle remains constant with a unity slope and **(E)** diverges with a greater than unity slope. APD giving unstable waxing oscillations. **(F)** Transient instabilities in the form of (i) early and (ii) delayed afterdepolarizations in the course and following full recovery of the AP waveform [**(A–E)** from Figure 14.4 of the work of Huang (2021)].

*Temporal electrophysiological heterogeneities* are typically observed as alternating variations in AP amplitude or APD between successive beats. Such *alternans* classically presages the breakdown of the regular pattern of electrophysiological activity and appearance of clinical (Nearing et al., 1991; Rosenbaum et al., 1994; Armoundas et al., 1998) or experimental arrhythmias (Pastore et al., 1999). Alternans in APD at a given heart rate, and therefore BCL, is thought to arise from variations in the timecourse of AP recovery or restitution to the resting potential, quantifiable as the APD. This variation, in turn, affects the subsequent diastolic interval (DI) prior to the AP corresponding to the following beat (Nolasco and Dahlen, 1968; Hayashi et al., 2007). The DI, thus, defines the period over which the membrane potential of its preceding AP is repolarized to its resting level. This recovery time, in turn, potentially affects the recovery properties, specifically the APD, of the subsequent AP. For example, shortened recovery times, DI, may preclude full recovery of the ion channel activity that underlies AP generation prior to the following beat. Typically, reductions or increases in DI have been reported to result, respectively, in reductions or increases in the APD of the subsequent AP. This effect follows a relationship termed the A-curve (Figure 4A) (Huang, 2021). However, at a fixed BCL, the resulting variations in APD would, in turn, alter the succeeding DI (Figure 4B). This latter effect is described by a linear D-line given by DI = BCL–APD (Figure 4C). These effects together would drive an interaction between DI and APD through successive cardiac cycles. Thus, the altered DI would, in turn, cause alterations in the APD of the subsequent AP and its DI that follows. This interaction can be described by superimposing the respective A-curve and D-lines.

The intersection between the A-curve and D-lines defines the steady-state APD and DI at any given BCL (Figure 4, Figures 4Da, Ea) (Sabir et al., 2008b). However, the interaction between the processes that they represent will vary with the heart rate as the latter, in turn, alters the BCL. Alterations in BCLs shift the D-line (Figures 4Db, Eb). This, in turn, alters the intersection point between the A-curve and D-line and, therefore, the DI corresponding to this intersection. The heart will then transition to a new APD as determined by the projection of this point to the A-curve (Figures 4Dc, Ec). This, in turn, alters the DI of the succeeding AP as given by the subsequent projection to the D-line (Figures 4Dd, Ed). The result is an iterative cycle of oscillating APD through successive heartbeats determined by the alternating projections to and from the A-curve and D-line.

Whether these oscillations converge, resulting in stable, persistent activity, or diverge, giving rise to unstable activity, is determined by the slope of the A-curves around the intersection region. For example, *zero* A-curve slope at the intersection results in an immediate attainment of a final steady-state APD and DI without oscillations. Where the A-curve slope falls *between zero and unity* (Figure 4D), the successive projection lines map a convergence back to the set point, giving a transient alternans ultimately stabilizing with a waning in the amplitude of the oscillation. The *unity slope* corresponding to the critical DI, DI_crit_, gives projection lines that neither converge nor diverge, yielding stable alternans. Intersections where *the A-curve slope exceeds unity* result in diverging projection lines. This corresponds to a progressively increasing instability and a waxing oscillation in which the projection lines ultimately veer away from the left-hand limit of the A-curve. This causes a pro-arrhythmic conduction block and potential re-entry (Figure 4E) (Matthews et al., 2010; Matthews et al., 2012; Matthews et al., 2013).

Finally, *intrinsic instabilities* in the AP waveform itself or following its full recovery to resting potential, if large enough to reach re-excitation threshold, potentially cause isolated triggered beats and, in the presence of arrhythmic substrate, persistent arrhythmia (January and Riddle, 1989). In the ventricles, *early afterdepolarizations* (EADs) interrupting AP recovery timecourses reflect events continuing from the ventricular phase 2 plateau (Figure 4Fi). Transient, *delayed afterdepolarizations* (DADs) follow full repolarization (Figure 4F(ii)) (Killeen et al., 2008a). Such abnormal triggering in the pulmonary or the superior caval veins may precipitate AF.

## 3 Ion channels underlying normal and abnormal rhythmic activity

### 3.1 Ion channels mediating action potential excitation

Membrane-level physiological processes can each be identified with specific surface membrane ion channels and transporters, each constituting disease and potential clinical therapeutic targets. Ion channels function and interact through both their sensitivity to and effects on membrane potential, constructing the electrophysiological events found in normal activity or disease states. Membrane transporters mediating metabolically coupled transport or ion exchanges also exert electrogenic effects dependent on the relative stoichiometry and charges of their translocated ions. These targets variously participate in activating rapid depolarizing (phase 0) (exemplified for human atrial (A) and human and murine ventricular (B) cardiomyocytes in Figures 2Aa, Ba) as well as recovery, early repolarizing (phase 1), plateau and late repolarization (phases 2 and 3), and electrically diastolic phases (phase 4) stages of AP waves (Figures 2Ab, Bb).

First, overall *cardiac pacing* is normally driven by SAN and in heart block, AVN and Purkinje tissue automaticity. Among inward *pacemaker currents* (Huang et al., 2016) are inward, hyperpolarization-induced cyclic-nucleotide-activated channel (HCN)-mediated *I*_f_ and electrogenic 3Na^+^/1Ca^2+^ exchange (NCX) currents driven by sarcoplasmic reticular (SR) Ca^2+^ release (Lakatta et al., 2010; Donald and Lakatta, 2023). This results in a time-dependent phase 4 depolarization from the background resting potential. This process is modulated by autonomic, adrenergic, or cholinergic stimulation or inhibition. It results in the membrane potential attaining the Ca^2+^ (*I*_Ca_) and then the Na^+^ current (*I*_Na_) excitation thresholds. This initiates the SAN AP (Lei et al., 2007). Clinical automaticity abnormalities arise from abnormal ionic current activity or altered background diastolic or resting potentials. They can manifest as sinus node disorder (SND), abnormal AVN or Purkinje tissue pacing, and spontaneous impulses in pathologically partially depolarized atrial and ventricular muscle.

Second, *Na* ^*+*^ *channels* typified by Nav1.5, activated by prior depolarization, carry rapidly developing inward depolarizing transmembrane *I*_Na_. This drives the rapid depolarizing activating phase 0 of the consequent atrial or ventricular cardiomyocyte APs. Nav1.5 shows complex functional and distribution patterns among subcellular cardiomyocyte subdomains; cardiomyocytes may further express additional Nav1.x subtypes (Remme, 2023). Subsequent *I*_Na_ inactivation contributes to AP recovery alterable by pathological persistent, potentially pro-arrhythmic, late, *I*_NaL_, currents (Chadda et al., 2017; Yu et al., 2018; Liu et al., 2023). Nav1.5 is also involved in SAN pacemaking through AP propagation from SAN to surrounding atrial muscle (Lei et al., 2005). Genetic loss or gain of *I*_Na_ function causes distinct pro-arrhythmic human Brugada (BrS) and long QT3 syndromes (LQTS3), respectively, experimentally recapitulated in loss (Martin et al., 2010; Martin et al., 2011b) or gain of Nav1.5 function murine models affecting AP activation and recovery (Killeen et al., 2008a; Sabir et al., 2008a). Both experimental and clinical BrS and LQTS3 aberrations and their arrhythmic tendency were accentuated/relieved by flecainide; quinidine produced the reverse effects (Benhorin et al., 2000; Stokoe et al., 2007; Martin et al., 2011b; Sabir et al., 2013). Nav1.5 mutations may also be involved in the impaired cardiac pacemaking in SND (Lei et al., 2005).

Third, similarly inward and depolarization-activated Cav1.2-mediated *I*_Ca_ may initiate APs in the SAN and AVN besides prolonging atrial and ventricular phase 2 plateau activation. Their loss-of-function mutations, recently exemplified by *CACNB2b-S143F* and *CACNA1C-G37R* (Zeng et al., 2023), resemble those involving Nav1.5 with pro-arrhythmic phenotypes accompanied by J wave syndromes (Huang, 2017).

Fourth, diverse, similarly depolarization-activated *voltage-dependent K* ^*+*^ *channels* contrastingly carrying repolarizing outward currents (Smith and Cain, 2004; Tamargo et al., 2004; Nerbonne and Kass, 2005; Enyedi and Czirjak, 2010; Schmitt et al., 2014; Huang, 2017; Jeevaratnam et al., 2017) contribute to AP recovery. Transient outward Kv4.3 and Kv4.2, *I*_to_, mediate early phase 1 repolarization and, along with atrial-specific Kv1.5 (*KCNA5*) carrying ultra-rapid *I*_Kur_ acetylcholine-sensitive GIRK1 and GIRK4 carrying *I*_KACh_, shorten atrial relative to ventricular APDS. Gain-of-function Kv4.3 and Kv4.2 mutations are implicated in AF (Alrabghi et al., 2023). Ventricular Kv11.1 (HERG or *KCNH2*) produce rapidly activating phase 1 rapid *I*_Kr_ and then inactivate through AP phases 0–2 (Sanguinetti and Tristani-Firouzi, 2006; Vandenberg et al., 2012) but re-activate over phase 3 and early phase 4 to terminate the plateau. *I*_Kr_ may actually peak during terminal repolarization phases of normally paced AP waveforms, potentially suppressing pro-arrhythmic actions of early afterdepolarizations or premature beats (Lu et al., 2001; Vandenberg et al., 2006). Kv7.1 (*KCNQ1*)-mediated *I*_Ks_ contrastingly activates more slowly over phase 2 producing a persistent phase 3 K^+^ conductance.

Fifth, *voltage-independent inward rectifying K* ^*+*^, Kir2.1, Kir2.2, and Kir2.3 (*KCNJ2*, *KCNJ12*, and *KCNJ4*), *channels* produce a reduced inward *I*_K1_ at the phases 0–2 depolarized voltages but outward current with phase 3 repolarization. Along with background K_2P_2.1 (*KCNK2*)-mediated K_2P_ currents and normally small adenosine triphosphate (ATP)-sensitive Kir6.2 (*KCNJ11*)-mediated *I*_KATP_ (Foster and Coetzee, 2016), they also stabilize phase 4 diastolic resting potentials. *Loss* and *gain* of K^+^ channel function involving *I*_Kr_, *I*_Ks_, and *I*_K1_ can produce pro-arrhythmic *long* (LQTS) and *short* QT syndrome (SQTS), both predisposing to atrial and ventricular arrhythmias (Hancox et al., 2023). Similarly, protein expressional changes recently exemplified in *I*_Ks_ were associated with ventricular arrhythmias. The ubiquitin-like-modifier leukocyte antigen F-associated transcript 10 (FAT10) may reduce Kv7.1 ubiquitination, decreasing *I*_Ks_ (Chen et al., 2023).

### 3.2 Ion channels mediating action potential conduction

AP propagation between successive cardiomyocytes underlying coherent waves of excitation (King et al., 2013a; Huang et al., 2020) depends on local circuit current flow driven by *I*_Na_ through low-resistance (Verheule et al., 1997) gap junctions (GJs) comprising connexins Cx40 and/or Cx43 (Moreno et al., 1994; Traub et al., 1994; Rohr, 2004). It is determined by maximum AP depolarization rates (d*V*/d*t*)_max_ dependent on *I*_Na_, membrane capacitance, and cytosolic and connexin resistances (Jeevaratnam et al., 2011; King et al., 2013a). Adjacent cardiomyocytes are joined end-to-end at specialized perinexal regions including closely apposed adjacent cell membranes (Rhett and Gourdie, 2012; Rhett et al., 2013; Veeraraghavan et al., 2014) with locally elevated Cx expression and Nav1.5 densities (Veeraraghavan et al., 2015; Hichri et al., 2018; Struckman et al., 2021) increased by Na_v_1.5 clustering (Salvage et al., 2020b). Mathematical modeling suggests such structural organization is additionally or alternatively compatible with a direct cell-to-cell ephaptic propagation of excitation (Sperelakis and Mann, 1977; Sperelakis, 2002; Mori et al., 2008; Carmeliet, 2019; Morris et al., 2024). An albeit slower propagation of excitation persists even with compromised gap junction coupling under pathological conditions following ischemic insult (Rohr, 2004), atrial fibrillation (Chaldoupi et al., 2009), and experimental loss-of-function Cx43 (Eloff et al., 2001; Gutstein et al., 2001) or Cx40 genetic platforms (Hagendorff et al., 1999; Bagwe et al., 2005). Both propagation mechanisms are potentially modifiable by the longer-term tissue fibrotic or inflammatory processes discussed below (Jeevaratnam et al., 2011).

## 4 A hierarchy of mechanisms for ion channel modification

**FIGURE 5:**
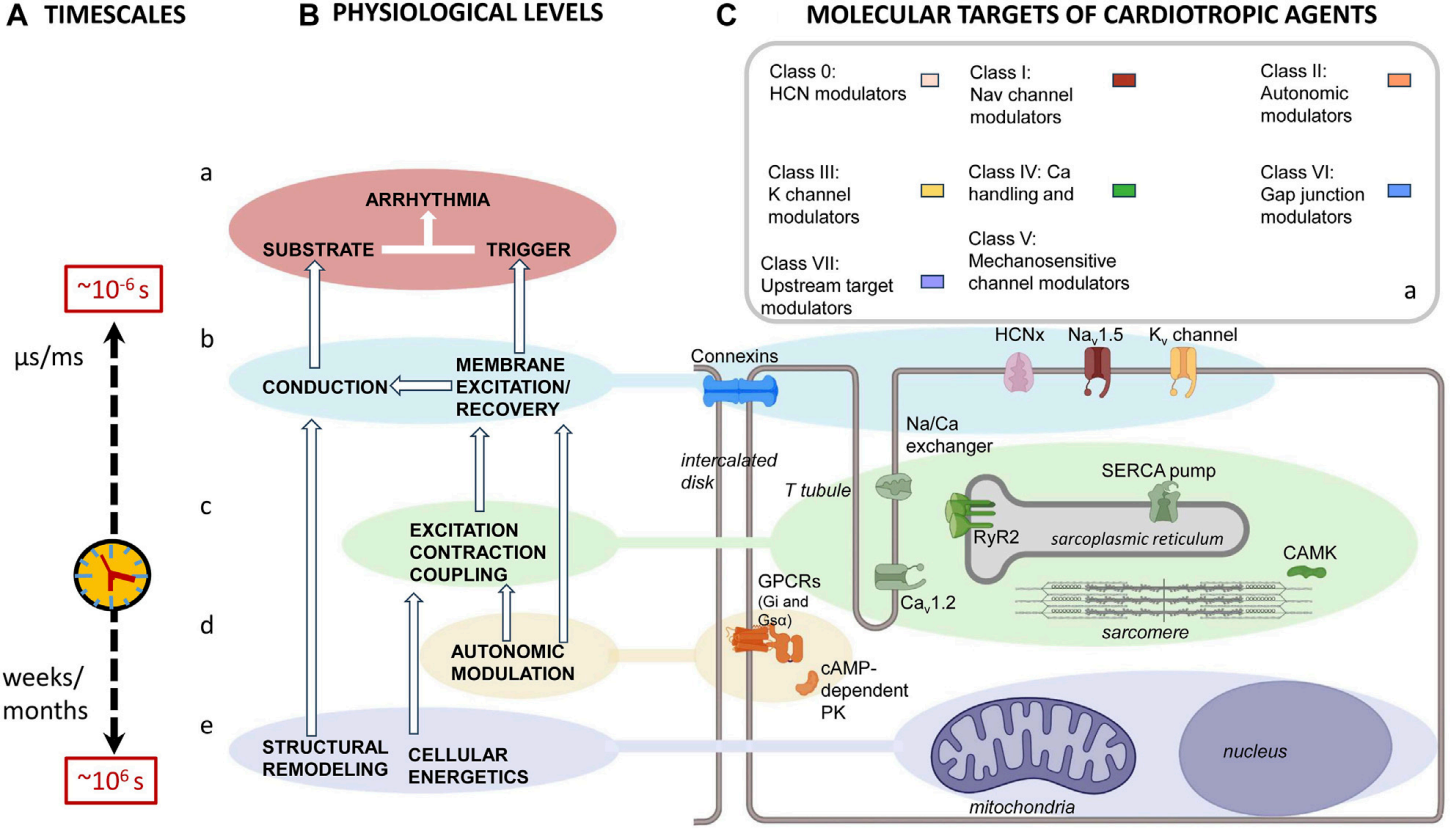
Mapping of physiological mechanisms, molecular targets, and therapeutic agents related to arrhythmia. **(A)** Pro-arrhythmic processes taking place over successively longer timescales running from ns/μs, ∼10^–6^ s timescale, molecular events, through ms/s events within each cardiac cycle or multiple cardiac cycles, to hours/days or even week/month, ∼10^6^ s, timescales resulting from **(B)** an interleaving hierarchy of physiological processes involving interactions (a) at the molecular/membrane level, interactions between surface membrane ion channels underlying automaticity and AP excitation, propagation and recovery. (b) At the cellular level, excitation contraction coupling processes mediate Ca^2+^ signaling. (c) At the systems level, autonomic inputs and their related G-protein signalling, modulated by central nervous system circadian rhythms. (d) Longer-term electrophysiological and structural remodeling effects related to metabolic feedback and other upstream modulators. These map onto **(C)** recently reclassified (a) anti-arrhythmic drugs that, in turn, act upon (b–e) the molecular targets mediating these physiological processes. These include (b) surface membrane ion channels contributing inward depolarizing hyperpolarization-activated cyclic nucleotide gate (HCN); cardiac Na^+^ (Nav1.5) and Ca^2+^ (Cav1.2) channel, outward K^+^ channel (Kvx.x), and conducting connexin (CX) mediated currents. (c) Excitation contraction coupling-related Cav1.2, cardiac ryanodine receptor type 2 (RyR2) and transient receptor potential channel (TRPx), and their regulatory calcium/calmodulin kinase II (CaMKII); (d) autonomic signaling through inhibitory Gi, and stimulatory G proteins, Gs, involving PKA signaling. Longer-term modulation involves (e) energetic and modeling processes.

The immediate cause of pro-arrhythmic events involves actions of, and interactions between, cell membrane ion channels within individual or between adjacent cardiomyocytes. However, these are influenced by a hierarchy of mechanisms. These extend over a 12-decade, logarithmic timescale. This ranges from ns/μs (∼10^–6^ s) timescales shown by molecular events, through cellular events extending over ms/s within each or multiple cardiac cycles, to hours/days or even weeks/months (∼10^6^ s) shown by remodeling processes (Figure 5A). Thus, there are (Figure 5B) (a) *cell surface membrane* ion channels and transporters underlying automaticity and AP excitation, propagation, and recovery and the (b) c*ellular* feedforward and feedback effects of excitation–contraction coupling and its triggering by Ca^2+^. These latter overlap over microsecond/millisecond time scales. In contrast, the (*c*) s*ystems-level* G-protein signaling-dependent autonomic inputs and their related central nervous system circadian rhythms extend over second/minute/hour time scales. Finally, (d) longer-term *upstream mechanisms* related to metabolic feedback and other upstream modulators extend over days/weeks/months. Nevertheless, each of these processes can be grouped (Figure 5C) by their participating biomolecules (Figure 5D) (Lei et al., 2018). The scheme complements and prompts extensions of existing modeling attempts that extend to full electromechanical coupling at the systems level. These had integrated local and transmural ventricular myocyte surface electrical (ten Tusscher et al., 2004) with the cytosolic and SR Ca^2+^ transport and storage properties outlined below. They also modeled the consequent Ca^2+^–troponin binding to cross-bridge cycling activity and myofilament mechanics (Rice et al., 2008). These finally extended to mechanical activity in realistic two- and three-dimensional ventricular cardiac tissue models (Pathmanathan and Whiteley, 2009; Adeniran et al., 2013; Huang, 2015).

Many clarifications of these relationships arise from monogenically modified murine models permitting single cardiomyocyte, tissue, whole organ, and systems-level experimental study (Huang, 2017). Murine hearts translate to human hearts in their two-sided atrial and ventricular circulations, pacing, or conducting SAN, AVN, and atrioventricular (AV) components. They differ in size, heart rates, some ion channel types (Figures 2Ba, b), and consequent detailed AP waveforms (Figures 2c, d). Nevertheless, they share major, depolarization and conduction, AP features and regional heterogeneities (Huang, 2017). Particular genetic variants recapitulate corresponding human clinical arrhythmic and pharmacological phenotypes. Genetically induced pluripotent stem cell-derived cardiomyocyte (iPSC-CM) platforms also show promise as cellular rather than systems models in not recapitulating *in vivo* conducting, Purkinje, and contractile tissue organization. However, many human-induced pluripotent stem cell-derived cardiomyocytes (hiPSC-CMs) show immature embryonic-like rather than adult atrial/ventricular phenotypes (Lee et al., 2012). Nevertheless, recent reports describe hiPSC-CMs with atrial AP properties and acetylcholine-activated K^+^ current expression (Ahmad et al., 2023). There are recent iPSC models for normal and disease changes in ion channel expression, Ca^2+^ homeostasis, neurocardiac interactions, and hypertrophy (Li et al., 2023; Chen et al., 2023; Langa et al., 2023; Zhou et al., 2023). Theoretical reconstructions remain useful in predicting physiological end effects (Alrabghi et al., 2023; Hancox et al., 2023). Ultimate translatability of such results still requires direct human clinical electrophysiological studies with recently innovated electrocardiographic (Van Der Werf and Lambiase, 2021) and electrical mapping capacities (Taggart et al., 2022).

## 5 Ion channel modulation at the level of the cell membrane

A number of associated proteins modify function and expression of surface membrane ion channels in short local feedback loops. First, auxiliary *β-subunits* modulate function in their various associated Na^+^, Ca^2+^, and K^+^ channel subtypes. Na^+^ channel β-subunits (Navβ) each comprise an amino-terminal immunoglobulin (Ig) connected to a single transmembrane domain and a short largely disordered intracellular region. They enhance channel trafficking to the surface membrane modifying peak *I*_Na_, modulate steady-state and kinetic inactivation (Namadurai et al., 2015), and ion channel clustering in the plasma membrane (Salvage et al., 2020).

The Navβ_1_ and Navβ_3_ isoforms associate non-covalently with α-subunits, mostly with the Ig domain binding to the domain III voltage-sensing module (VSM); the Navβ_2_ and Navβ_4_ Ig domains covalently associate via a disulfide bond to an extracellular loop region of domain II (Salvage et al., 2020a). However, Nav1.5 constitutes an interesting exception to this rule. Here, the Navβ_1_ and Navβ_3_ subunit Ig domains cannot bind to domain III VSM because the binding site is likely blocked by glycosylation. Similarly, Navβ_2_ and Navβ_4_ Ig domains cannot form disulfide bonds because the necessary free cysteine residues are absent in the Nav1.5 α-subunit. Consequently, Nav1.5-associated β-subunit Ig domains may be free to form extended cis and trans cross-links. Here, Navβ_1_-subunits may interact in *trans* influencing cell proximity. In contrast, cis-Navβ_3_ interactions may enhance local clustering of Nav1.5 (Salvage et al., 2020a). These may enhance ephaptic cell-to-cell AP conduction at intercalated disc perinexal membranes (Salvage et al., 2020a; Salvage et al., 2023). Genetic β-subunit mutations produce pro-arrhythmic phenotypes resembling conduction defects shown by *Scn5a* ± arrhythmic models (Hakim et al., 2008; Hakim et al., 2010).

Of K^+^-channel-associated β-subunits, *KCNE2*-encoded minK-related protein 1 (MiRP1) is associated with many K^+^ channel α-subunits. It modifies steady-state and kinetic activation and deactivation properties of HERG K^+^ channel-mediated *I*_Kr_ (Lu et al., 2003). Similarly, *KCNE1*-encoded β-subunits are associated with I_Ks_ channels. Their genetic ablation causes abnormal AP recovery, increasing APDs and inverting transmural APD gradients (Thomas et al., 2007; Hothi et al., 2009), and pro-arrhythmic phenotypes including early afterdepolarizations (EADs) and spontaneous VT in murine hearts recapitulating human Jervell and Lange–Nielsen syndrome (LQTS5) (Balasubramaniam et al., 2003).

Second, ion channels interact with cell adhesion, signal transduction, and cytoskeletal anchoring proteins. Nav1.5 interacting proteins may also function in specific subcellular Nav1.5 localizations producing regional variations in Nav1.5 expression, *I*_Na_, density, and kinetics between subcellular microdomains. At lateral cardiomyocyte membranes, Nav1.5 interacts with dystrophin–syntrophin complexes, calcium/calmodulin (CaM)-dependent serine protein kinase, and caveolin-3. At intercalated disks, Nav1.5 associates with N-cadherin, connexin-43, β_IV_-spectrin, and desmosomal proteins including plakophilin-2 and desmoglein-2. Gene mutations involving some of these proteins are associated with arrhythmic disorders suggesting Nav1.5 dysfunction (Shy et al., 2013; Remme, 2023).

## 6 Feedforward and feedback between excitation–contraction coupling and surface membrane processes

Excitation–contraction coupling refers to the feedforward events connecting Nav1.5-mediated surface and transverse (T-) tubular membrane depolarization by AP initiation and propagation to initiation of contraction. Tubular Cav1.2 channels sense triggering surface membrane voltage changes, transducing these into ryanodine receptor (RyR2)-mediated SR Ca^2+^ store release by Ca^2+^-induced Cav1.2-RyR2 coupling (Endo, 2009). This likely occurs at T-SR junctional regions where the relevant membranes assume geometrically close ∼100–400 nm parallel alignment while remaining electrically separate (Martin et al., 2003; Dulhunty, 2006). Nav1.5, Cav1.2, and RyR2 come into close proximity within the resulting restricted diffusion spaces permitting local ion accumulation or depletion (Bardsley et al., 2021). Local Ca^2+^ release might then significantly elevate [Ca^2+^]_TSR_ (Saucerman and Bers, 2012; Despa et al., 2014; Sanchez et al., 2021), in turn elevating bulk cytosolic [Ca^2+^]i-activating troponin, thereby triggering myofilament action and cardiomyocyte contraction. The background cytosolic [Ca^2+^]_i_ is ultimately restored by SR Ca^2+^-ATPase (SERCA2), which mediates the Ca^2+^ return to its SR store and expulsion into the extracellular space by sarcolemmal NCX and, to a lesser extent, sarcolemmal Ca^2+^-ATPase. Further intracellular organelles, including lysosomes and mitochondria, are often regulated by further signaling molecules, such as cADP-ribose, nicotinic acid adenine dinucleotide phosphate (NAADP), and inositol tris-phosphate (IP_3_), which also modulate cellular Ca^2+^ homeostasis (Terrar, 2023).

**FIGURE 6:**
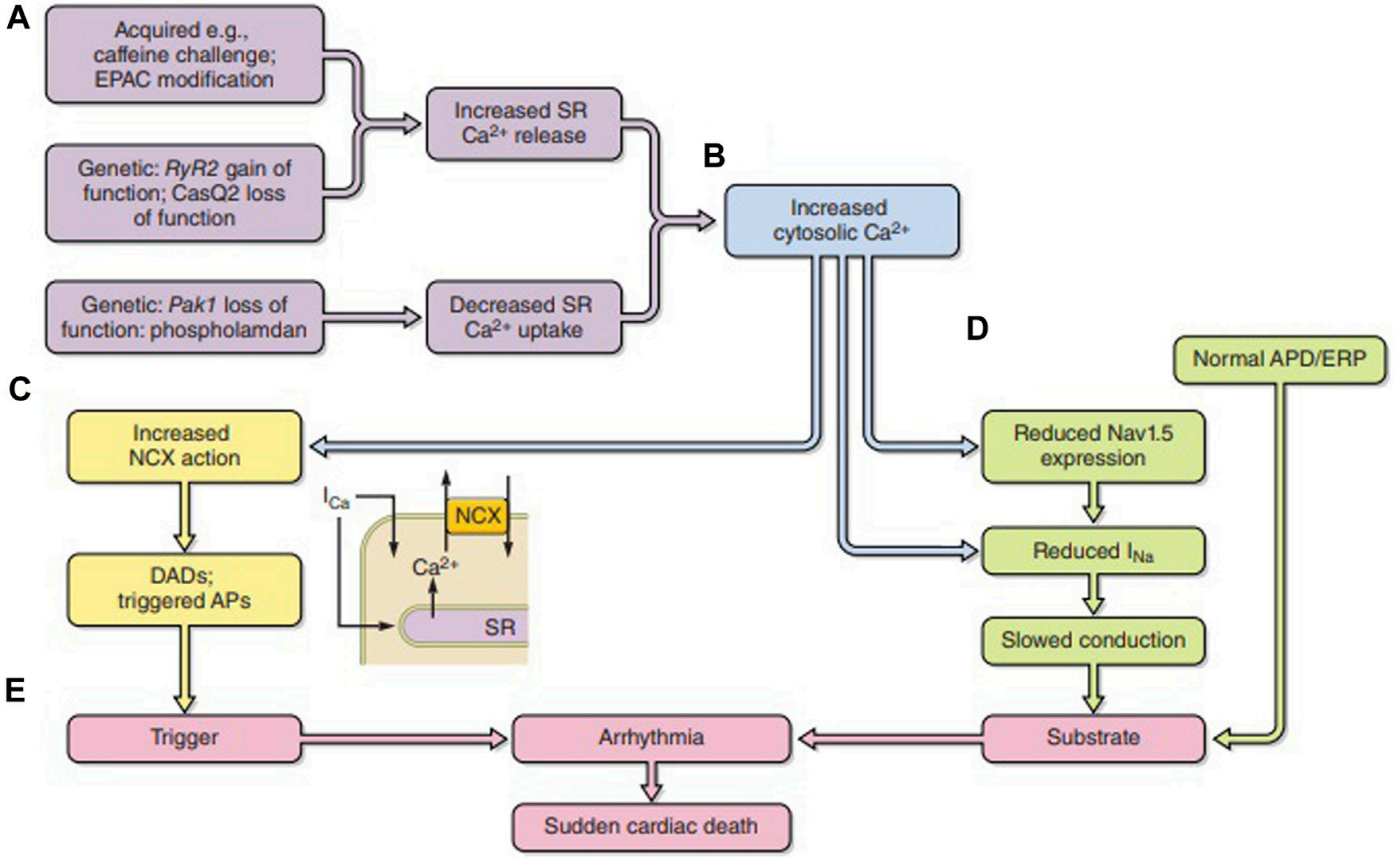
Pro-arrhythmic feedback relationships between excitation contraction coupling and surface membrane ionic channel function. **(A)** Acquired or genetic perturbations increasing sarcoplasmic reticular (SR) Ca^2+^ release or decreasing SERCA-mediated Ca^2+^ reuptake both alter cytosolic [Ca^2+^] **(B)**. This alters **(C)** 3Na^+^/1Ca^2+^ exchange (NCX) electrogenic activity causing triggering DADs. It may also **(D)** reduce Nav1.5 synthesis or membrane trafficking or directly alter Nav1.5 function, slowing down AP conduction. The trigger/substrate combination **(C, D)** causes **(E)** sustained arrhythmia [from Figure 18 of the work of Huang (2017)].

Feedback actions of consequently altered intracellular Ca^2+^ fit its strategic second messenger function through widespread cellular processes and cell types (Figure 6A) (Huang, 2017). Released Ca^2+^ itself exerts a short-loop slow RyR inactivation (Laver and Curtis, 1996). Further longer-loop feedback modulation may modify surface membrane channel or transporter activity with pro- or anti-arrhythmic effects (Figures 6B–D), acting on either arrhythmic triggering or substrate (Figure 6E). Of voltage-sensitive channels mediating inward depolarizing current, Na^+^ and Ca^2+^ channel C-terminal domains contain both direct and indirect Ca^2+^ regulatory binding sites in the form of one or more EF hand motifs and an isoleucine–glutamine (IQ) CaM-binding domain, respectively. Their III–IV loops also contain Ca^2+^-CaM-binding sites. The channels additionally possess multiple kinase, including CaM kinase II (CaMKII) and phosphokinase C (PKC), and phosphorylatable regulatory sites. One or more of these sites have been implicated in Ca^2+^-mediated feedback *inhibition* of Nav1.5 in intact cardiac myocytes and implicated in arrhythmogenic phenotypes in both pharmacological (King et al., 2013b; Valli et al., 2018b) and genetic disease models (King et al., 2013c; Zhang et al., 2013; Salvage et al., 2021; Salvage et al., 2023). Similar mechanisms may operate for skeletal muscle Nav1.4 (Matthews et al., 2019; Sarbjit-Singh et al., 2020; Liu et al., 2021). Ca^2+^, likely through CaMKII, conversely increases *I*_NaL_ with potential pro-arrhythmic effects (Liu et al., 2023).

Of *ion exchange processes*, NCX stoichiometry produces inward, potentially pro-arrhythmic *I*_NCX_ under conditions of elevated [Ca^2+^]_i_ in polarized membranes (Shattock and Bers, 1989).

Of voltage-sensitive channels mediating outward hyperpolarizing current, Ca^2+^-activated K^+^ channel opening contrastingly hyperpolarizes, thereby reducing membrane excitability. In addition to membrane depolarization, large conductance Big K^+^ (BK) channel opening requires μM local [Ca^2+^] binding to two distinct high-affinity intracellular C-terminal domain Ca^2+^-binding sites (Zeng et al., 2005; Yang et al., 2015; Sancho and Kyle, 2021), modifying sino-atrial node firing rate and cardiac pacing (Meredith et al., 2014; Pineda et al., 2021). Voltage-insensitive small conductance (SK) SK1, SK2, and SK3 K^+^ channels (Adelman et al., 2012; Weisbrod, 2020) respond to RyR-induced [Ca^2+^]_i_ through a regulatory C-terminal CaM-binding domain (Neelands et al., 2001; Li et al., 2023). SK2 channels, in particular (Schmitt et al., 2014), are clinically implicated in atrial fibrillation (Healey et al., 2005) and arrhythmia and hypertrophy accompanying clinical and experimental ventricular failure (Terentyev et al., 2014; Gu et al., 2018). SK2 modified AP waveform in pro-hypertrophic Pak-1-deficient murine experimental platforms predisposing to ventricular hypertrophy and failure following angiotensin II challenge (Yang et al., 2021). Finally, of anion channels, Ca^2+^-activated Cl^−^, TMEM16A, channels are [Ca^2+^] sensitive (Yang and Colecraft, 2016).

## 7 Autonomic feedforward and feedback

Autonomic, sympathetic and parasympathetic, modification of pacing, ion current activation, and excitation–contraction coupling involves transmitter and co-transmitter binding to G-protein-coupled receptors (GPCRs). G-protein-linked activation cascades show significant amplification through their constituent signaling molecules exerting multiple inotropic, chronotropic, and lusitropic effects on cardiac function (Bers, 2002). Both levels of and balance between sympathetic and parasympathetic activity may affect arrhythmogenicity: autonomic dysregulation is involved in multiple cardiac arrhythmias. Thus, both increased sympathetic and vagal rebound activity amplify ventricular arrhythmic risk. Its modulation may offer novel therapeutic options.

The *sympathetic* transmitters adrenaline and noradrenaline are, respectively, released into the circulation by adrenal medullary release and locally by widely distributed cardiac sympathetic nerve terminals. They, respectively, preferentially bind surface membrane β_1_- and β_2_-adrenergic receptors. Cardiomyocytes express β_1_-adrenergic receptors, activating the stimulatory G_s_ protein. Its G_α_ subunit then binds guanosine triphosphate (GTP), dissociates from the GPCR and its βγ-subunit, and activates adenylyl cyclase, enhancing cellular cyclic 3′,5’ adenosine monophosphate (cAMP) levels, with diverse compartmented cellular actions (Zaccolo, 2023). Of nerve–cardiomyocyte communication mechanisms under current study, ventricular *Dbh* ^*+*^ catecholaminergic cardiomyocytes (*Dbh* ^*+*^ Cate-CMs) expressing dopamine β-hydroxylase occur in close structural relationship with the sympathetic innervation. Their catecholaminergic-type vesicles suggest endocrine functions. They may also be involved in embryonic development and maturation of the cardiac conduction system (Wang et al., 2017).

At the *membrane* level, sympathetic activity increases heart rate and modifies AP generation and waveform. cAMP binds to and opens SAN HCN, increasing *I*_f_ and heart rates. It also activates protein kinase A (PKA) whose widespread phosphorylation actions excite Nav1.5, Kv11.1, Kv7.1, and Cav1.2, increasing rapid inward *I*_Na_ and slow outward *I*_Kr_ and *I*_Ks_ and I_CaL_, increasing ventricular AP amplitudes but shortening the AP plateau durations. It also accelerates SAN pacemaker potentials (Huang and Lei, 2023). In *excitation–contraction coupling*, sympathetic activity increases *I*_CaL_ and net cellular Ca^2+^ entry increasing rates and force of subsequent muscle contraction. PKA-mediated RyR2 phosphorylation also reduces regulatory FKBP12 ligand binding otherwise stabilizing RyR2 closure, thereby increasing RyR2 Ca^2+^ sensitivity and Ca^2+^-induced Ca^2+^ release. It also phosphorylates phospholamban (PLN), relieving its SERCA2 inhibition, enhancing diastolic SR Ca^2+^ store.

The Epac2 isoform of cAMP-dependent exchange protein activates CaMKII (Takla et al., 2020; Terrar, 2023) and, therefore, RyR2-mediated SR Ca^2+^ release (Hothi et al., 2008). At *further upstream levels* discussed below, Epac1 activates Ca^2+^-dependent calcineurin signaling, driving hypertrophic, morphological, and cytoskeletal changes. Additionally, excitatory postganglionic sympathetic co-transmitters, adenine nucleotides, act on metabotropic P2Y receptors. Adenosine (A_1_) receptor activation activates phosphokinase C (PKC), which also acts on voltage-gated Na^+^ and K^+^ channels, L-type Ca^2+^ channels, and RyR2. Finally, sympathetic responses can differ among cardiomyocyte types, exemplified by the distinct pulmonary vein (PV) and left atrial cardiomyocyte responses to noradrenaline implicated in atrial ectopy (Bond et al., 2020).

The *parasympathetic*, inhibitory, transmitter, acetylcholine (ACh), acts on muscarinic (M_2_) receptors activating the G_i2_ protein in the SAN, AVN or atria, in both the presence and absence of, pre-existing adrenergic challenge. It does so in ventricular tissue in the presence of such challenge. Its G_α_ subunit binds GTP and splits off from the receptor and its Gβγ-subunit. The latter opens GIRK1 and GIRK4 channel components, increasing inward rectifying I_KACh_ or I_KAdo_, particularly in supraventricular tissue (Schmitt et al., 2014; Dascal and Kahanovitch, 2015; Lerman, 2015). The dissociated G_iα_ inhibits adenylate cyclase (AC) and, therefore, cAMP production (DiFrancesco, 1993), increasing *I*_CaL_ and *I*_f_ in pacemaker cells. G_i_ activation also increases protein phosphatase (PP2A) activity through cell division control protein 42 homolog (Cdc42)/Ras-related C3 botulinum toxin substrate 2 (rac2) and the cardioprotective p21-activated kinase PAK1. PP2A dephosphorylates PKA-phosphorylated proteins at the same serine/threonine phosphorylation sites, reversing their L-type Ca^2+^ channel, RyR2 and PLN effects. Parasympathetic action, thus, slows heart rates and decreases contractile force.

PAK1 may be cardioprotective through increasing PP2A activity (Ke et al., 2009; Wang et al., 2015) and its remodeling actions (Liu et al., 2011; Wang et al., 2014; He et al., 2023; Jung et al., 2023). It may also modify Cav1.2/Cav1.3 (*I*_CaL_)-mediated Ca^2+^ entry, RyR2-mediated SR Ca^2+^ release, and CaMKII-mediated SERCA2a and NCX transcriptional regulation (He et al., 2023) and promote SERCA activity (Ke et al., 2009; Wang et al., 2014; Wang et al., 2015). PAK1 deficiency may promote atrial arrhythmogenesis under adrenergic stress conditions through posttranslational and transcriptional modifications of key molecules including RyR2 and CaMKII (Jung et al., 2023).

Over longer *systems*-level timescales, autonomic innervation provides effector pathways for central nervous responses to environmental inputs. Normal circadian ion channel remodeling cycles explain SAN pacemaking changes, resulting in higher background heart rates during wakefulness than during periods of sleep (Jesus et al., 2021). These are likely driven by sympathetic, as opposed to parasympathetic, actions coupled to suprachiasmatic nuclear circadian rhythms. Rather than variations of autonomic transmitter activity (Black et al., 2019), recent evidence attributes these to periodic transcriptional cardiac remodeling cycles varying ion channel abundances and their consequent ionic current densities. The SAN ion channel transcriptome for many important cardiac ion channels, particularly HCN, displays circadian rhythms (Anderson et al., 2023; Black et al., 2019; D’Souza et al., 2021; Wang et al., 2021), sensitive to chronic but not acute pharmacological autonomic blockade (Pitzalis et al., 1996; Black et al., 2019). It possibly involves cAMP response element action on key *Per1* and *Per2*.18 clock genes (Travnickova-Bendova et al., 2002). Critically needed are future studies of autonomic plasticity and age-related properties (Chadda et al., 2018; Takla et al., 2023).

## 8 Upstream modulatory targets: energetic feedback

Longer-term pro-arrhythmic upstream cellular energetic and tissue remodeling can accompany metabolic pathology in various conditions, including acute hypoxic/ischemia–reperfusion (Liu et al., 2020), chronic obesity, insulin resistance and type 2 diabetic conditions (Vianna et al., 2006; Sato et al., 2009; Kucharska-Newton et al., 2010), and cardiac failure, accompanying primary hypertrophic or fibrotic changes (Terentyev et al., 2008; Grivennikova et al., 2010; Belevych et al., 2012; Chouchani et al., 2014). First, maintenance of ionic gradients and Ca^2+^ cycling processes represents ∼30–40% of cardiomyocyte ATP utilization. Of the latter, ∼90% is replenished by their extensive mitochondrial network (Leone and Kelly, 2011; Nguyen et al., 2019; Wang et al., 2019).

**FIGURE 7:**
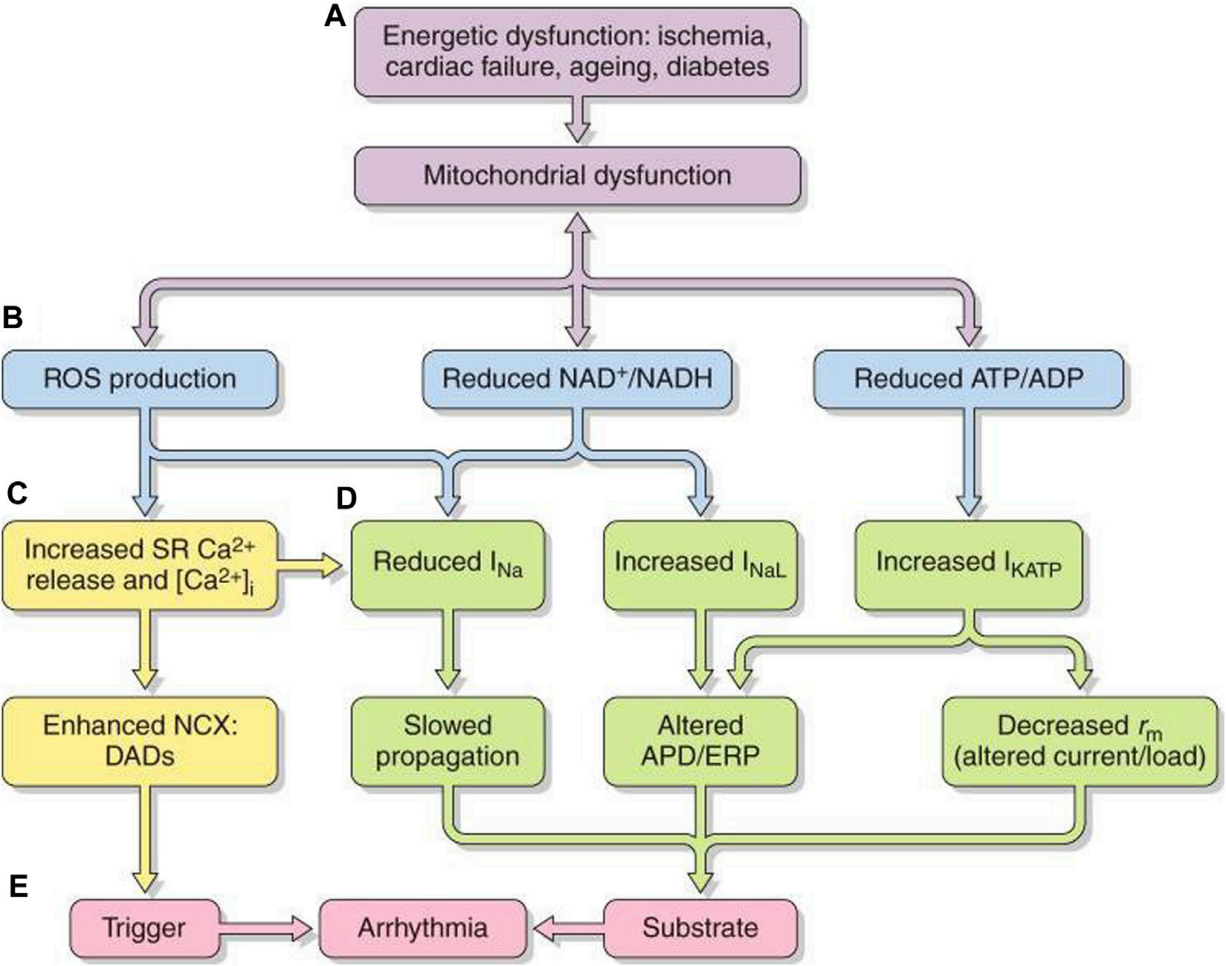
Pro-arrhythmic feedback relationships between energetic dysfunction and surface membrane ionic channel activity. Simplified scheme in which **(A)** energetic dysfunction in ischemia, cardiac failure, aging, or diabetes is reflected in **(B)** mitochondrial dysfunction causing ROS production, altered NAD^+^/NADH, and ATP/ADP. This leads to increased **(C)** RyR2-mediated SR Ca^2+^ release and increased [Ca^2+^]_i_ NCX and consequent DAD triggering activity. **(D)** Altered Na^+^ and K^+^ channel function affects AP excitation, propagation, and recovery. The trigger/substrate combination **(C, D)** causes **(E)** sustained arrhythmia [from Figure 20 of the work of Huang (2017)].

Mitochondrial mass and function are regulated by transcriptional coactivators (Sonoda et al., 2007): peroxisome proliferator-activated receptor (PPAR) γ coactivator-1 (PGC-1) family members are highly expressed in oxidative tissues including the heart. Of these, mitochondrial promoter PGC-1α or PGC-1β expression increases with upstream signals including cold exposure, fasting, and exercise, likely signaling anticipated cellular energy requirements (Figure 7A) (Huang, 2017). PGC-1 proteins exert matching multi-level regulation of cellular mitochondrial function and metabolism. Of their activated nuclear receptor targets, PPARα regulates genes involved in mitochondrial fatty acid oxidation; estrogen-related receptor alpha (ERRα) regulates mitochondrial fatty acid oxidative and oxidative phosphorylative energy transduction pathways (Vega et al., 2000). Additionally, PCG-1α coactivation of nuclear respiratory factor-1 (NRF-1) and -2 (NRF-2) (Wu et al., 1999) modulates nuclear-encoded transcription factor Tfam expression required in replicating, maintaining, and transcribing mitochondrial DNA (Garesse and Vallejo, 2001) and mitochondrial biogenesis (Vega et al., 2000; Huss et al., 2004). These mechanisms may underlie the induction of nuclear genes encoding mitochondrial proteins in energetic pathways, including the tricarboxylic acid cycle, and nuclear and mitochondrial genes encoding electron transport chain and oxidative phosphorylation complex components in cultured cardiomyocytes with forced PGC-1 expression (Lehman et al., 2000). PCG-1s fall in obesity, insulin resistance, type II diabetes mellitus, and aging in parallel with mitochondrial dysfunction (Leone and Kelly, 2011; Dillon et al., 2012).

Mitochondrial dysfunction, whether intrinsic or arising from excessive energetic demand, compromised vascular oxygen supply or pathological energetic disorders, destabilizes inner membrane potentials, driving their electron transport chain. At the *cell membrane level,* the consequently compromised ATP and/or rising adenosine diphosphate (ADP) increase sarcolemmal K-ATP (sarcKATP) channel opening probabilities (Akar and O’Rourke, 2011). This shortens APDs and ERPs and compromises cell excitability and AP propagation (Akar and O’Rourke, 2011). These events predispose to re-entrant arrhythmia (Fosset et al., 1988; Faivre and Findlay, 1990).

Increased reactive oxygen species (ROS) production accompanying oxidative stress also influences cardiomyocyte excitability (Figures 7B–D). The resulting atrial and ventricular arrhythmic tendency may result from reduced Nav1.5 expression (Liu et al., 2010), increased *I*_NaL_(Liu et al., 2023), and reduced connexin-43 (Cx43) trafficking and function (Akar and O’Rourke, 2011; Yang et al., 2014; Yang et al., 2015) and cell–cell coupling (Smyth et al., 2010) and inhibited voltage-dependent *I*_K_ (Wang et al., 2004), sarcolemmal K_ATP_ (Weiss et al., 1989), and *I*_Ca_. These effects could affect AP conduction (Liu et al., 2010) and underlie the atrial ERP shortening and AF initiation with rapid pacing (Figure 7E) (Carnes et al., 2001; Dudley et al., 2005). Right atrial appendages of AF patients show increased oxidative stress markers (Emelyanova et al., 2016). Accompanying changes in reduced (NADH) or oxidized nicotinamide adenine dinucleotide (NAD^+^), reflecting cell oxidative state, also, respectively, inhibit and enhance activity even with normally expressed Nav1.5 (Liu et al., 2010).

At *the excitation–contraction coupling level*, increased ROS oxidize RyR2, increasing SR Ca^2+^, and reduce SERCA-mediated Ca^2+^ reuptake, increasing cytosolic [Ca^2+^]_I_ (Kukreja et al., 1988). CaMKII oxidation may induce a kinase activity similar to auto-phosphorylated CaMKII (Erickson et al., 2008). ROS also oxidize and activate PKA (Eager and Dulhunty, 1998). ROS, thus, altered intracellular Ca^2+^ cycling (Terentyev et al., 2008; Brown and O’Rourke, 2010; Bovo et al., 2012) in aging rabbit ventricular myocytes, effects reversed by mitochondrial specific ROS scavengers (Terentyev et al., 2008). At the *level of further upstream targets*, ROS are implicated in fibroblast activation and transforming growth factor-β (TGF-β) production, leading to cardiac fibrosis (Friedrichs et al., 2012).

Mice deficient in either but not both *PCG-*1α or *PGC-*1β survive to adulthood. *Pgc-1α*−/− hearts had normal baseline function but failure with increased afterload (Arany et al., 2005). *Pgc-1β*−/− hearts showed normal background features but blunted heart rate responses to adrenergic challenge (Lelliott et al., 2006), increased arrhythmic propensity, and APD alternans and VT episodes (Gurung et al., 2011). Single cells showed altered ion channel expression and spontaneous diastolic Ca^2+^ transients. Chronic studies in young (12–16 weeks) and aged (>52 weeks) *Pgc-1β*−/− mice suggested sinus node and AVN dysfunction in intact animals following β_1_-adrenergic challenge (Ahmad et al., 2018b). The atria and ventricles of Langendorff-perfused *Pgc-1β*−/− hearts showed increased arrhythmic inducibility with age, accompanied by reduced (d*V*/d*t*)_max_, increased AP latencies, and reduced APD, together reducing λ and increasing arrhythmogenicity (Ahmad et al., 2017; Valli et al., 2017a; Ahmad et al., 2018a). These findings correlated with direct demonstrations of reduced *I*_Na_ but not *I*_K_ (Valli et al., 2018a; Ahmad et al., 2019). *Pgc-1β*−/− hearts also showed accelerated age-related fibrotic change, discussed further in the next section (Ahmad et al., 2017; Valli et al., 2017c).

## 9 Upstream cardiac remodeling

Second, longer-term inflammatory and structural, fibrotic and hypertrophic, changes can pro-arrhythmically add to and influence the physiological processes above (Nattel et al., 2007; Dobrev and Nattel, 2010; Iwasaki et al., 2011). They involve inflammatory cell recruitment, cellular proliferation, angiogenesis, and extracellular matrix accumulation (Murphy et al., 2015). Accumulating evidence implicates inflammatory processes involving tumor necrosis factor (TNF)-α, macrophage migration inhibitory factor, interleukin (IL)-1β, IL-6, and JUN N-terminal kinases (JNKs) with pro-inflammatory M1 macrophages participating particularly in AF. Additionally, pro-inflammatory (M2) macrophages may enhance atrial fibroblast proliferation and differentiation. TNF-α, in turn, activates profibrotic transforming growth factor-β (TGF-β) signaling and matrix metalloproteinase secretion. TGF family members, particularly TGF-β1, regulate tissue homeostasis and repair, immune and inflammatory responses, extracellular matrix (ECM) deposition, and cell differentiation and growth (Yang et al., 2010). TGF-β1 is a key contributor to fibrosis (Dobaczewski et al., 2011). TGF-β1 signaling is itself activated by angiotensin II (Ang II) action on local cardiac renin–angiotensin system (RAS) angiotensin receptor type 1 ATR_1_s, which themselves directly activate TGF-β production.

TGF-β likely acts through Smad and synergistically with extracellular signal-regulated protein kinase ½ (ERK1/2), c-Jun NH_2_-terminal kinase (JNK), p38 mitogen-activated protein kinases (p38MAPK), all mitogen-activated protein kinases (MAPK) (Dobaczewski et al., 2011). It stimulates myofibroblast differentiation, key to the fibrotic process, and ECM protein synthesis (Desmouliere et al., 1993) through inhibiting matrix metalloproteinase (MMP) and inducing tissue inhibitor metalloproteinase (TIMP) synthesis (Mauviel, 2005). Ang II similarly acts in this, both itself, in synergy with TGF-β1 (Schnee and Hsueh, 2000; Murphy et al., 2015), and itself activating TGF-β1 signaling (Schultz et al., 2002; Dobaczewski et al., 2011). TGF-β1 administration-induced fibroblast proliferation causes atrial fibrosis (Dzeshka et al., 2015; Hu et al., 2015) and AF (Lifei et al., 2020).

ATR_1_s also activate G protein, Gα_q/11_, Gα_12/13_, and G_βy_, and non-G protein-related pathways, modulating multiple oxidase and kinase signaling pathways. Among serine/threonine kinases, Ang II- or ROS-mediated CaMKII activation enhances phosphorylation of excitation–contraction coupling-related proteins and cell survival and transcription factors, driving hypertrophic and inflammatory gene expression (Swaminathan et al., 2012; Rusciano et al., 2019). PKC activates NAD(P) H oxidase, thence increasing ROS production and consequent cardiomyocyte hypertrophy (Nakamura et al., 1998; Sugden and Clerk, 1998). Activation of the MAPKs, including ERK1/2, JNK, and p38MAPK, is implicated in cell growth and hypertrophy (Wollert and Drexler, 1999), as well as cardiac fibrosis, through increased procollagen I, procollagen III, fibronectin, and TGF-β gene transcription. Epidermal growth factor receptor (EGFR) and platelet-derived growth factor (PDGF) insulin receptor signaling can also occur. Of non-receptor tyrosine kinases, Src, janus kinase/signal transducer and activator of transcription IL (JAK/STAT), and focal adhesion kinase (FAK) (Mehta and Griendling, 2007), JAK/STAT may promote pressure overload and ischemia-related hypertrophy (Booz et al., 2002). Finally, recent reports implicate upregulated Notch signaling in hypertrophic and dilated cardiomyopathy (Langa et al., 2023).

Cardiac remodeling first affects *surface membrane ion channel* function, *excitation–contraction coupling*, and conduction of excitation. Of inflammatory cytokines, TNF-α may reduce *I*_Ca,L_, while increasing *I*_to_, systolic Ca^2+^ transients, diastolic intracellular [Ca^2+^], and inward NCX and decreasing SR ATPase expression. These changes accentuate DAD-induced triggered activity (Lee et al., 2007). It may also alter Cx40 and Cx43 expression and distribution. JNK2 activates CaMKII, increasing diastolic SR Ca^2+^ leak, and downregulates Cx43 expression, compromising cell-to-cell communication and atrial AP conduction (Yan et al., 2018) Inflammasome NLRP3 activation upregulates RyR2 expression and CaMKII-dependent hyperphosphorylation and consequent pro-arrhythmic diastolic SR Ca^2+^ release, similarly inducing DADs and ectopic firing (Yao et al., 2018). It also increases *I*_Kur_ and *I*_KACh_ and increases atrial hypertrophy and fibrosis (Heijman et al., 2020). PAK1 signaling may exert protective signaling actions through Cav1.2/Cav1.3 (*I*_CaL_)-mediated Ca^2+^ entry, RyR2-mediated SR Ca^2+^ release, and CaMKII-mediated regulation of SERCA2a and NCX transcription (He et al., 2023). Its deficiency may modify posttranslational and transcriptional processing in the key Ca^2+^ homeostatic molecules RyR2 and CaMKII with atrial pro-arrhythmic effects under adrenergic stress (Jung et al., 2023).

Second, fibrotic changes may contribute to both AF and ventricular arrhythmias. Increased fibrosis-related gene expression accompanying altered surface and Ca^2+^ homeostasis ion channel gene expression may contribute to SAN dysfunction in experimental rat pulmonary arterial hypertension (Logantha et al., 2023). Disruption of myocardial bundle continuity and gap junction formation due to increased extracellular matrix associated with fibrotic and/or hypertrophic change reduces and accentuates heterogeneities in AP conduction velocity, disrupting AP propagation wavefronts and promoting re-entry (Davies et al., 2014). Connexin-mediated electrotonic coupling between cardiomyocytes and inexcitable fibroblasts with relatively depolarized (∼-30 mV) resting potentials may depolarize cardiomyocyte resting membrane potentials altering myocyte excitability, slow conduction, shorten APD, and induce spontaneous phase 4 depolarization (Nattel, 2018). Additionally, Ca^2+^ signaling in fibroblasts employs stretch-sensitive short TRPC3 or melastatin-type TRPM7 transient receptor potential (TRP) channels, both upregulated in AF fibroblasts (Yue et al., 2015).

Other downstream processes may feed forward to modify these changes. Both genetic Nav1.5 (Jeevaratnam et al., 2012; Jeevaratnam et al., 2016) and Pgc1-β-related metabolic deficiencies (Ahmad et al., 2017; Valli et al., 2017b; Ahmad et al., 2018a; Ahmad et al., 2018b) were associated with accentuated TGF-β1-mediated fibrosis, possibly driving observed electrophysiological remodeling underlying age-related SND dysfunction (Hao et al., 2011) and pro-arrhythmic atrial and ventricular fibrosis. The former non-canonical roles for Nav1.5 in cellular biological processes extend to associations with morphological changes in dilated and arrhythmic cardiomyopathies (Remme, 2023).

## 10 Mechanistic insights translated into novel anti-arrhythmic therapies

Pharmacological intervention underpins much clinical arrhythmia management. Both modern drug innovation and optimizing existing therapeutic strategies require systematically classified mechanisms of action at identified molecular physiological targets and their rational correlation with single cell, tissue, or organ tissue effects and, in turn, clinical indications and therapeutic actions. This requirement had been addressed by successive historic Singh–Vaughan Williams (VW) (Vaughan Williams, 1975) and updated European Society of Cardiology classifications (Task force of the working group on arrhythmias The European Society of Cardiology, 1991). The most recent modernized classification scheme (Lei et al., 2018) responding to the recent cardiac electrophysiological and pharmacological insights outlined here pragmatically retained but extended the original VW classes. It mapped actions on molecular targets within more recently characterized levels of surface membrane ion channel, excitation–contraction coupling, autonomic function, and longer-term energetic and remodeling changes outlined above. The resulting scheme for cardiac arrhythmic mechanisms thereby offered a basis for understanding existing explorations for novel arrhythmic therapies (Lei et al., 2018), and reviewing drug cardiac safety (Saadeh et al., 2022).

Of the introduced novel drug categories, *class 0*, bearing on cardiac automaticity, includes the use-dependent SAN inhibitor ivabradine. It reduces heart rate by acting on *I*_f_, sparing myocardial contractility and vascular tone, actions distinct from the existing VW classes II and IV. It is clinically approved for reduced ejection fraction under circumstances of cardiac failure, adjunct therapy improving clinical outcomes by reducing heart rate. It is yet to be established for stable ischemic heart disease. It may also benefit patients with inappropriate sinus tachycardia intolerant of class II or IV agents (Koruth et al., 2017).

Of extended VW classes I–IV, *class I* now includes class Id drugs acting on *I*_NaL_ this complements the existing Class Ia–c, which reduce early *I*_Na_ thereby reducing AP phase 0 slopes and overshoots, and modify APD and ERP. Class Ia drugs classically bind to the Nav1.5 open state with τ ∼1–10 s dissociation time constants. They concomitantly block *I*_K_, slow AV conduction, and increase ERP and APD. Classes Ib and Ic contrastingly bind preferentially to the inactivated state with rapid τ ∼0.1–1.0 s or slow τ > 10 s dissociation. This results in a use-independent and use-dependent channel block and a slowed AV conduction. These insights clarify some of flecainide’s paradoxical actions. Thus, flecainide exerts anti-arrhythmic actions in gain of Na^+^ channel, particularly *I*_NaL_, function associated with LQTS3 and experimental murine Scn5a^+/Δkpq^. In contrast, it shows pro-arrhythmic actions under post-infarct conditions and clinical and experimental *Scn5a*+/− Brugada syndrome (Martin et al., 2010; Martin et al., 2011a). Of drugs in the new *class Id*, ranolazine produces a frequency-dependent block of the pro-arrhythmic *I*_NaL_. This shortens APD and increases refractoriness and repolarization reserve in LQTS3, pathological bradycardic and ischemic conditions, and cardiac failure (Belardinelli et al., 2015). Intracellular Na^+^ overload, otherwise arising from such *I*_NaL_, increases reverse-mode NCX, promoting pro-arrhythmic intracellular Ca^2+^ overload and SR Ca^2+^ leak. Ranolazine may also reduce atrial peak *I*_Na_ and Kv11.1-mediated *I*_K_ (Burashnikov et al., 2007; Antzelevitch et al., 2011).

A broadened *class II* encompassed further modifiers of G-protein-mediated signaling besides the β-adrenergic antagonists originally used to slow down SAN pacing and AVN conduction. These included new selective β1-(atenolol and bisoprolol) and non-selective β-adrenergic antagonists (carvedilol), adenosine A_1_-activators (adenosine), and cholinergic muscarinic M_2_ receptor inhibitors (scopolamine) and activators (carbacholine and pilocarpine) (Singh, 2004). Future possibilities could arise from numerous (∼150) further orphan GPCRs (Lei et al., 2018). Finally, centrally acting sympathetic inhibitors, such as monoxidine, have been investigated in relation to AF management (Hu et al., 2023).

An expanded *class III* encompassed drugs directed at the large number of more recently discovered, sometimes atrial or ventricular-specific, voltage-sensitive and -insensitive K^+^ channel types. This added to the voltage-gated K^+^ channel blockers originally employed to delay AP phase 3 repolarization, lengthening ERPs. Exemplars, including investigated agents directed at atrial arrhythmia, included those directed at voltage-sensitive *I*_K_ such as the atrial *I*_Kur_, including XEN-D0103 (Ford et al., 2016) and vernakalant, for rapid AF (Wettwer et al., 2013), with the latter producing some *I*_Na_ and *I*_NaL_, and *I*_KACh_ in addition to *I*_Kur_ block. New I_Kr_ blockers under development include vanoxerine (Lacerda et al., 2010). Investigational agents directed at voltage-insensitive *I*_K_ include the SK channel inhibitor AP30663 (Bentzen et al., 2020) and the K2P blocker doxapram (Wiedmann et al., 2022).

Major advances in excitation–contraction coupling have yielded significant progress in *class IV drugs* beyond the L-type Ca^2+^ channel inhibitors introduced to reduce SAN and AVN rates and conduction (Vaughan Williams, 1975). First, investigational non-selective and phenylalkylamine and benzothiazepine inhibitors supplement dihydropyridine Cav1.2 and Cav1.3 surface membrane *I*_CaL_ and I_CaT_ inhibitors.

Second, of agents targeting intracellular membrane molecules mediating SR Ca^2+^ release and reuptake, the experimental drug dantrolene enhances CaM-RyR2 binding and, consequently, N-terminal-central domain interactions, stabilizing RyR2 closed states (Pabel et al., 2020). Recent reports implicate flecainide in class IV RyR2 open-state block reducing RyR2 open probabilities, in addition to its class I actions. Both experimental and clinical reports support its application in catecholaminergic polymorphic ventricular tachycardia (Watanabe et al., 2009; Hilliard et al., 2010; Hwang et al., 2011; Van Der Werf et al., 2011; Salvage et al., 2018). As expected from concentration dependences for the latter effect (Watanabe et al., 2009; Galimberti and Knollmann, 2011; Heath et al., 2011), flecainide (1 µM) was, respectively, pro- and paradoxically anti-arrhythmic in murine WT and homozygotic *RyR2-P2328S* atria. In WT, it reduced *I*_Na_, slowing conduction velocity, sparing refractory periods. Contrastingly, in *RyR2-P2328S*, it restored normal WT *I*_Na_ values, similarly sparing refractory periods. Dantrolene challenge yielded similar results. These findings suggest its rescue of an arrhythmic phenotype in *RyR2-P2328S* involves relief of Nav1.5 inhibition by reducing RyR2-mediated Ca^2+^ release in general feedback effects of Ca^2+^ homeostasis on different Nav subtypes (Salvage et al., 2018; Salvage et al., 2022; Salvage et al., 2023). Higher flecainide concentrations (5 µM) reduced *I*_Na_ in both WT and *RyR2-P2328S* (Salvage et al., 2015).

Carvedilol has similar dual class IV in addition to β-adrenergic blocking class II actions: its antioxidant actions reduce RyR2 phosphorylation and oxidation, and its open-state channel block decreases RyR2-mediated Ca^2+^ release (Zhou et al., 2011). Finally, explorations of SERCA activation by istaroxime also have potential translational implications (Ferrandi et al., 2013; Huang, 2013).

Third, continued progress in understanding Ca^2+^ homeostasis demonstrates novel regulatory novel Ca^2+^ signaling molecules. In addition to CaMK-II inhibitors, exemplified by RA608 (Mustroph et al., 2020), and investigational PAK1-targeted agents (He et al., 2023) influencing phosphorylation of cytosolic handling proteins, further signaling molecules, including cADP-ribose, nicotinic acid adenine dinucleotide phosphate (NAADP) (Terrar, 2023), and Epac proteins (Li et al., 2017; Valli et al., 2018b), offer potential investigational targets.

The modernized scheme additionally introduced novel classes V–VII. Investigational drugs from *class V* blockers of mechanically sensitive TRP channels included *N*-(p-amylcinnamoyl) anthranilic acid, which may also target Cl^−^ channels (Harteneck et al., 2007), the TRPC3 inhibitor pyrazole-3 (Harada et al., 2012), and TRPM7 inhibitor, LBQ657 (Lifei et al., 2020). These may additionally potentially inhibit hypertrophic and fibrotic changes (Harada et al., 2012; Seo et al., 2014). *Class VI* targeting cell–cell electrotonic coupling includes the gap junction blocker carbenoxalone (De Groot et al., 2003) and Cx43 opening peptide rotigaptide (Hsieh et al., 2016). *Class VII* targeting upstream signaling and remodeling contains significant representatives for its component processes. Among inflammatory cytokines, these inhibit TNFα decoy receptor [etanercept (Aschar-Sobbi et al., 2015)], IL-1β [colchicine (Wu et al., 2020)], and IL1 [anakinra (De Jesus et al., 2017)]. Microtubule disruption by colchicine may prevent NLRP3 inflammasome assembly (Wu et al., 2020). Profibrotic modeling is targeted by the TGF-β1 inhibitor pirfenidone (Lee et al., 2006) and TGF-β1 receptor inhibitor GW788388 (de Oliveira et al., 2012). Agents targeting further signaling pathways include the ERK-phosphorylation inhibitor mesalazine (Künzel et al., 2021) and the HMG-CoA reductase inhibitors, statins (Adam et al., 2011). PAK1 activators may inhibit maladaptive, pro-arrhythmic, hypertrophic remodeling and progression in cardiac failure (Tsui et al., 2015; Wang et al., 2018; He et al., 2023) in addition to its role regulating ion channel activity, Ca^2+^ homeostasis, and cardiac contractility (Ke et al., 2009; Wang et al., 2014; Wang et al., 2015). Angiotensin-converting enzyme and angiotensin receptor blockers, aldosterone receptor antagonists, are further exploratory targets (Savelieva and Camm, 2008; Geng et al., 2020; Saljic et al., 2022; Hu et al., 2023).

## 11 Discussion: future therapeutic prospects

Research in the physiological sciences has long involved work in successive, mutually reinforcing, interacting cycles between laboratory and clinic. Identified clinical or novel physiological phenomena initiate the development of representative experimental physiological models. These insights, in turn, prompt innovative clinical testing, management, and treatment, themselves prompting further iterations of experimental testing to identify novel physiological targets, investigational new drugs, and interventions (Huang et al., 2020; Huang and Lei, 2023). Future such cycles could add immunotherapeutic approaches, including antibody-based agents with further enhanced target specificities even targeting particular channel isoforms (Presta, 2008) and micro-RNAs/shRNAs knocking down protein expression at the mRNA level provided suitable delivery methods become available (Chakraborty et al., 2017). Within a framework of a modernized drug classification, these could themselves add to understanding arrhythmic events and their modification besides facilitating future clinical development.
